# Solar irradiation levels during simulated long‐ and short‐term heat waves significantly influence heat survival, pigment and ascorbate composition, and free radical scavenging activity in alpine Vaccinium gaultherioides

**DOI:** 10.1111/ppl.12686

**Published:** 2018-03-13

**Authors:** Matthias Karadar, Gilbert Neuner, Ilse Kranner, Andreas Holzinger, Othmar Buchner

**Affiliations:** ^1^ Institute of Botany, Functional Plant Biology University of Innsbruck 6020 Innsbruck Austria

## Abstract

In the 20th century, annual mean temperatures in the European Alps rose by almost 1 K and are predicted to rise further, increasing the impact of temperature on alpine plants. The role of light in the heat hardening of plants is still not fully understood. Here, the alpine dwarf shrub Vaccinium gaultherioides was exposed in situ to controlled short‐term heat spells (150 min with leaf temperatures 43–49°C) and long‐term heat waves (7 days, 30°C) under different irradiation intensities. Lethal leaf temperatures (LT_50_) were calculated. Low solar irradiation [max. 250 photosynthetic photon flux density (PPFD)] during short‐term heat treatments mitigated the heat stress, shown by reduced leaf tissue damage and higher F_v_/F_m_ (potential quantum efficiency of photosystem 2) than in darkness. The increase in xanthophyll cycle activity and ascorbate concentration was more pronounced under low light, and free radical scavenging activity increased independent of light conditions. During long‐term heat wave exposure, heat tolerance increased from 3.7 to 6.5°C with decreasing mean solar irradiation intensity (585–115 PPFD). Long‐term exposure to heat under low light enhanced heat hardening and increased photosynthetic pigment, dehydroascorbate and violaxanthin concentration. In conclusion, V. gaultherioides is able to withstand temperatures of around 50°C, and its heat hardening can be enhanced by low light during both short‐ and long‐term heat treatment. Data showing the specific role of light during short‐ and long‐term heat exposure and the potential risk of lethal damage in alpine shrubs as a result of rising temperature are discussed.

**Abbreviations**
a.s.l.: above sea level
Aq. dest.: distilled water
A_tot_: total ascorbate
DHAA: dehydroascorbate
DMF: dimethylformamide
DPPH: 2,2‐diphenyl‐1‐picrylhydrazyl
DTT: dithiothreitol
EDTA: ethylenediaminetetraacetic acid
*F*_v_/*F*_m_: potential quantum efficiency of photosystem 2
HHCs: heat‐hardening chambers
HHM: half hourly mean
HSP: heat shock proteins
HTTS: heat tolerance testing system
LHC: light‐harvesting complex
MHCs: mobile heating chambers
NEM: N‐ethylmaleimid
NPQ: non‐photochemical quenching
PPFD: photosyntetic photon flux density
PSII: photosystem 2
PTOX: plastoquinol terminal oxidase
QS: quantum sensor
ROS: reactive oxygen species
sHSP: small heat shock proteins
TE: Trolox equivalents
TEM: transmission electron microscopy
Trolox: 6‐hydroxy‐2,5,7,8‐tetramethylchroman‐2‐carbonacid
VAM: visual assessment method
VEM: visual estimation method
WR: working reagent

## Introduction

In the 20th century, global mean surface temperatures rose by 0.55 K [Note: temperatures are presented in °C and temperature differences in Kelvin (K) according to Leuzinger et al. 2010], while in the European Alps mean temperatures rose by almost 1 K (Haeberli and Beniston 1998, Böhm et al. 2001). Predictions for further increases in global temperature range from 0.3 to 4.8 K for the time period from 1998–2012 to 2100 (IPCC 2013), with recurrent short‐term heat spells as well as long‐term heat waves. As a consequence, changes in species composition and plant migration toward higher altitudes have been reported for almost all major mountain ranges in Europe (Grabherr et al. 1994, Pauli et al. 2003, 2012, Erschbamer 2007, Kudernatsch et al. 2008, Lenoir et al. 2008, Steinacher and Wagner 2012, Ladinig et al. 2015), and this trend is expected to continue.

Many alpine plant species have evolved prostrate growth forms which have been suggested to confer competitive advantage by prolonging the growing season (Larcher 1980, Körner and Cochrane 1983). However, on calm, sunny days prostrate growth can occasionally be deleterious due to critical overheating (Larcher and Wagner 1976, Gauslaa 1984, Neuner et al. 1999, Buchner and Neuner 2003, Körner 2003). In addition, short‐term heat spells during midday may coincide with low night time temperatures, resulting in leaf temperature amplitudes of up to 50 K (Körner and Larcher 1988) with heating rates of up to 22.5 K h^−1^ (Neuner and Buchner 2012). Therefore, fast stress responses (Kranner et al. 2010) and in particular adjustments of heat tolerance by heat hardening (Alexandrov 1977, Gauslaa 1984) are necessary to ensure survival. For example, within one single day, heat tolerance increased by 4.7 K in *Silene acaulis* from 48.6 to 53.3°C (Neuner et al. 2000), and by 9.5 K in *Minuartia recurva* from 45.4 to as high as 54.9°C (Buchner and Neuner 2003).

Exposure to high temperatures above species‐specific heat thresholds (typically above 30°C) is known to induce heat hardening (Alexandrov 1977, Neuner et al. 2000). Such heat exposure can increase the stability of photosystem II (PSII) (Schreiber and Berry 1977, Havaux and Tardy 1996) and induce readjustment of thermal response and temperature optimum of net photosynthesis (Larcher 1980).

Interestingly, heat treatments applied in the dark have been shown to trigger heat hardening (Gauslaa 1984, Havaux and Tardy 1996). However, it is not fully understood how illumination during heat treatment affects the heat hardening process, and data on the effects of light in combination with heat on heat hardening are controversial. For example, the combination of light and short‐term heat stress had a negative impact on cut off leaves of *Oryza sativa* (Yin et al. 2010). By contrast, under laboratory conditions, long‐term exposure of plants to heat and strong light enhanced heat hardening (Maier 1971), and short‐term heat exposure in the light increased heat stability of PSII (Schreiber and Berry 1977, Weis 1982, Havaux et al. 1991, Havaux and Tardy 1996). A purpose‐built ‘heat tolerance testing system’ (HTTS) was recently developed that allows for the measurement of responses in intact plants in situ under controlled conditions in the field, without removing plants from their natural habitat (Buchner et al. 2013). Using the HTTS, it was shown that light during short‐term heat exposure may have a positive effect on heat tolerance and photosynthetic performance (Buchner et al. 2013, 2015).

The combination of high irradiation intensities and heat can cause a rise in reactive oxygen species (ROS) leading to an increased activity in enzymatic and non‐enzymatic ROS‐scavenging mechanisms (Jaleel et al. 2009). The two major water‐soluble antioxidants, ascorbate and glutathione (Noctor and Foyer 1998, Foyer and Noctor 2011) are implicated in the protection of the photosynthetic apparatus in plants exposed to strong light and heat (Laureau et al. 2011). Additionally, zeaxanthin is a key player in the xanthophyll cycle, dissipating excess light energy (Demmig‐Adams and Adams 1996, Demmig‐Adams 2003). Zeaxanthin also seems to increase heat tolerance of PSII and the water splitting complex (Havaux 1998, Havaux et al. 2007, Jahns and Holzwarth 2012), and the stability of thylakoid membranes during heat stress (Tardy and Havaux 1997). Furthermore, plants exposed to a combination of heat and strong light produce more zeaxanthin than under strong light alone (Streb et al. 2003, Dongsansuk et al. 2013). Thus, there could be cross reactivity between the mechanisms of heat hardening and the ability to acclimate to high light intensities.

Adaptations of the thylakoid membranes are important in plant acclimation to changes in temperature (Raison et al. 1982, Hugly et al. 1989) and light (Leong and Anderson 1984). Temperatures between 31 and >35°C were reported to cause dismantling of grana stacks in *Hordeum vulgare* (Smillie et al. 1978) and *O. sativa* (Vani et al. 2001), respectively, and >42°C in *Ranunculus glacialis* (Larcher et al. 1997), and formation of heat shock granules in the cytoplasm was observed in *Vigna unguiculata* at 45°C (Dylewski et al. 1991). The latter consist of heat shock proteins (HSP) and small heat shock proteins (sHSP), which play a role in protecting proteins and mRNA against degradation during stress events (Forreiter et al. 1997, Larcher et al. 1997, Kirschner et al. 2000), increasing the likelihood of plants surviving during heat stress (Miroshnichenko et al. 2005).

In this study *Vaccinium gaultherioides* Bigelow, a woody dwarf shrub commonly found in the lower alpine zones, which have been associated with an increased risk of heat‐damage (Ladinig et al. 2015), was selected as the study species. Under field conditions, we tested the effects of light in combination with simulated short‐ and long‐term heat exposure on heat hardening. Different heat doses (intensities × duration) were combined with various irradiation levels. By including various irradiation levels we addressed the question whether there is a relationship between heat and irradiation and if the irradiation level matters. We also investigated whether heat hardening under different light intensities involves alterations in photoprotective mechanisms. We mimicked (1) typical alpine midday heat peaks by applying short‐term heat spells (43, 45, 47 and 49°C; 150 min) and (2) a heat wave by controlled long‐term heat exposure (>30°C for 7 days). We then studied the effects of different solar irradiation intensities, applied in the heat hardening phase, on heat tolerance of leaves, PSII, pigment composition, xanthophyll cycle activity, ascorbate redox state, free radical scavenging and chloroplast ultrastructure (the latter during long‐term heat exposure only). We hypothesized that the presence of light during both short‐term and long‐term heat exposure will increase leaf heat tolerance compared to heat exposure in the dark or under low light conditions. Furthermore, we aimed to assess the frequency of exposure to critically high leaf temperatures by monitoring leaf temperatures throughout the summer in a *V. gaultherioides* population in the field.

## Materials and methods

### Plant material and study site

*Vaccinium gaultherioides* Bigelow (Ericaceae) is a typical representative of the subalpine dwarf shrub heath on lime‐free acidic soils and nutrient‐poor grasslands of the European Alps between 1500 and 2700 m above sea level (a.s.l.). *Vaccinium gaultherioides* is deciduous and usually grows to a height of 10–30 cm. The study site was located within the timberline ecotone (1950 m a.s.l.; 47°12′N/11°27′E) on the north‐west facing slope of Mount Patscherkofel near Innsbruck, Austria. During the study period, first sunlight hit the study site at around 08:00 h and lasted until 19:30 h with no trees or buildings blocking out the sunlight.

### Microclimate

Micrometeorological data were recorded using a data logger (CR1000, Campbell Scientific, Loughborough, UK). Photosynthetic photon flux density (PPFD) was measured using a quantum sensor (QS, Delta‐T, Cambridge, UK). Thermocouple sensors (TT‐TI‐40, Omega Engineering Inc., Stamford, CT) were attached to the lower leaf surface of eight leaves of *V. gaultherioides* with an air‐permeable adhesive (3 M Transpore™, 3 M Österreich GmbH, Perchtoldsdorf, Austria). Data were recorded at 1 min intervals and afterward summarized to half hourly means (HHM).

### Controlled heat treatments

#### Short‐term heat spell treatment

Short‐term heat spells lasted for 150 min and were simulated in the field with the HTTS (Buchner et al. 2013) (Appendix S1 and S2). The system consists of a software‐controlled (based on LabView 2012, National Instruments, Austin, TX) central supply unit that supports eight cylindrical heat exposure chambers (25 cm high, ø 15 cm) made of translucent Plexiglas (XT 29070, Röhm, Darmstadt, Germany). Each exposure chamber is equipped with four mobile temperature sensors (TT‐TI‐40, Omega Engineering Inc., Stamford, CT) to measure actual leaf temperatures. Leaf target temperature is controlled by automated comparison of the measured mean leaf temperatures with the actual set temperature. Small ventilators ensure homogenous leaf temperatures during heat exposure. An air humidity of 100% produced by humidifiers prevents cooling of the leaves by transpiration.

Four exposure chambers of the system were operated in light mode, allowing ambient solar irradiation to pass freely through the Plexiglas and reach the plant samples inside the chambers. In contrast, four other chambers were operated in dark mode, using steel cylinders which were placed over the exposure chambers to prevent solar irradiation from reaching the plant samples.

The short‐term heat spell treatments were conducted on August 15, 2012. As irradiation intensity and temperature can draw a significant effect on heat tolerance of leaves, the heat treatment was started just before sunrise to exclude effects of variable preconditions in terms of irradiation and temperature. This should ensure maximum physiological homogeneity of the leaves, and came close to the natural situation as in the alpine zone leaf heating starts with sunrise and is most pronounced before midday. Each temperature treatment started with an initial temperature stabilization phase in which leaves were exposed to 30°C for 30 min (darkness). Thereafter, under slowly increasing natural solar irradiation conditions (maximum PPFD 110 μmol m^−2^ s^−1^) leaf temperatures were successively increased until reaching the target temperatures (43, 45, 47 and 49°C) within a 120‐min long linear heating phase. Hereafter, simulated heat‐spell temperatures and light conditions are abbreviated in the text by the target temperature (43, 45, 47 or 49°C) and by L for light mode and D for dark mode. The heating rates applied are close to naturally occurring heating rates that were measured in leaves of alpine plants before midday hours (Neuner and Buchner 2012). Immediately after the simulated short‐term heat spells, heat tolerance was determined in situ using the HTTS by exposing the already enclosed sample twigs for 30 min to selected target temperatures (43, 45, 47 and 49°C) in darkness (D) and under natural solar irradiation conditions (L) at maximum PPFD 250 μmol m^−2^ s^−1^. Chlorophyll fluorescence measurements [*F*_v_/*F*_m_ (potential quantum efficiency of photosystem 2)] were taken 6 h and 3 days after the heat spell treatment, always on five predetermined and tagged leaves. Leaf tissue damage was determined once, 3 days after the short‐term heat spell treatment, and corresponding LT_50_ values were calculated.

#### Long‐term heat wave treatment

Long‐term heat waves lasted for 7 days (June 30–July 6, 2012) and were conducted in the field with three special heat‐hardening chambers (HHCs) (Appendix S3 and S4). For simulation of long‐term heat waves, the HHCs were placed over natural stands of *V. gaultherioides* plants. The HHCs are rebuilt hotbeds (Juwel Hotbed ‘Easy Fix’, Imst, Austria; dimensions: 100 × 120 cm). For heating inside the HHCs six ceramic fan heaters (CE AT858, 150 W, 115 × 130 × 75 mm, Conrad, Hirschau, Germany) were attached to the HHCs roof. To prevent overheating six axial fans (ø 120 mm; FAN103, LogiLink, Schalksmühle, Germany) were fitted into the side windows of the HHCs for cooling with fresh air from outside. Heating fans and axial fans were controlled by a simple on/off regulation using a relay interface with Darlington driver stage (MKS3PI‐5 DC12, OMRON, Kyoto, Japan). The target mean leaf temperature was set to 30°C during daytime and 15°C during the night. In each HHC 16 thermocouple sensors (TT‐TI‐40 and GG‐TI‐28, Omega Engineering Inc., Stamford, CT) and a PPFD QS (Delta‐T) were installed and connected to a data logger/multiplexer combination (CR1000, AM25T, Campbell Scientific) for measurement of leaf temperatures, air temperature and light intensity. The temperature within each HHC was automatically regulated by comparing the target mean leaf temperature (daytime: 30°C) with the actual mean leaf temperature which was calculated at high time resolution (1 s – intervals) from the data of 15 thermocouples attached to the lower leaf surfaces by an air permeable adhesive (Transpore, BM‐Austria GmbH, Vienna, Austria).

At the end of the long‐term heat wave treatment for each HHC mean leaf temperature and mean PPFD over 7 days were calculated separately for the daytime and for the nighttime.

All plants were watered daily in order to prevent drought stress.

Three different light intensities were applied in three separately operated HHCs by shading with garden fleece (Windhager, Thalgau, Austria) and white linen that acted as neutral filters, which had been preliminarily tested by a spectrophotometer (Appendix S3). One HHC was shaded by two layers of garden fleece, resulting in a reduction of PPFD to 30% of natural light. Another HHC was shaded by four layers of garden fleece and was additionally covered with white linen. Inside of this HHC light intensity was reduced to 12% of natural light. One HHC was kept unshaded, resulting in 60% of the outside PPFD within the HHC. Simulated light intensities are abbreviated by PPFD60%, PPFD30% and PPFD12% throughout the text. Heating during the day began when the QS in HHC PPFD60% registered light intensities higher than 50 μmol photons m^−2^ s^−1^. Additionally, untreated plants growing in close proximity to the HHCs were investigated. They received full solar irradiation (100% PPFD) but also lower leaf temperatures and therefore do not represent a control sensu stricto. Fresh leaves were collected daily from PPFD100%, PPFD60%, PPFD30% and PPFD12% between 14:00 and 16:00 h and LT_50_ values were calculated daily.

### Determination of heat tolerance

To determine heat tolerance, leaves were directly exposed for 30 min to a set of different target temperatures. Thirty minutes is a commonly used time span for the determination of heat tolerance (Kreeb and Cernusca 1990, Larcher 1990). The set of target temperatures was selected so that the lowest temperature caused no damage (0%) whereas the highest temperature killed the leaves (100% damage). Temperature differences between target temperatures were chosen in 2 K steps (e.g. 41, 43, 45°C, etc.). Heat tolerance was either conducted on detached leaves ex situ with mobile heating chambers (MHC; Buchner and Neuner 2001) or on intact plants in situ with the HTTS (Buchner et al. 2013).

In both cases, percentage leaf injury caused by heat was assessed once after a latent period (short‐term heat spell treatment: 3 days, long‐term heat wave treatment: 24 h). Two different methods were used: (1) the visual assessment of leaf injury and (2) the assessment by chlorophyll fluorescence (*F*_v_/*F*_m_; the potential quantum efficiency of PSII). The dose‐to‐effect plot, i.e. the plot of heat exposure temperature vs heat damage (%) follows a classical logistic function. This function was fitted to the data using P‐Fit software (Fig. P 2.7, Biosoft, Durham, NC). One parameter of the calculated logistic function is the LT_50_, i.e. the temperature that causes 50% heat damage to the leaf that can be read from the curve‐fitting protocol. LT_50_ was calculated using *F*_v_/*F*_m_ (LT_50 *F*v/*F*m_) and visual assessment of leaf injuries (LT_50 visual_).

#### Ex situ heat exposure: MHC

On detached leaves, heat tolerance was determined using MHC (Buchner and Neuner 2001). The system consists of eight exposure chambers made of thin aluminum plates (200 × 150 × 2 mm) equipped with a thermocouple sensor (GG‐TI‐28, Omega Engineering Inc., Stamford, CT), heating mats at the outer surface and a Styrofoam coating inside and outside to prevent heat loss. Inside the heat exposure chambers temperature can be controlled with high precision (typically ±0.2 K; Buchner and Neuner 2001). Ten leaves of *V. gaultherioides* were each fixed to eight thermostable transparencies (15 × 10 cm) with a special adhesive (3 M Transpore, 3 M Österreich GmbH). Transparencies loaded with leaf samples were inserted into the exposure chambers. To prevent transpiration cooling of the leaves during heat exposure, leaves were sprayed with tap water before closing the exposure chambers. After all chambers were loaded with leaf samples, 30 min heat exposure at target temperatures was initiated (38–52°C in 2 K steps). After the end of the heat treatment, leaves were placed on wet tissue paper inside plastic bags. Assessment of viability was conducted after storage of samples for 24 h at room temperature under dim light (PPFD <40 μmol m^−2^ s^−1^).

#### In situ heat exposure: HTTS

In the field, plants were heat treated using the HTTS as described in detail by Buchner et al. (2013). Eight twigs of *V. gaultherioides*, similar in shape and appearance, bearing approximately the same number of leaves were inserted into one heat exposure chamber each. The chambers were closed using special lids containing slashes, so that the stems of the twigs can be passed through the lids while still attached to the roots, without injury. Four chambers were operated in dark mode and the other four in light mode. Five leaves per exposure chamber were selected and labeled with pieces of differently colored drinking straws wrapped around the leaf stalks. Only these leaves were monitored in the viability assays. To prevent damage to the very thin and fragile leaves of *V. gaultherioides* thermocouples were attached by magnetic leaf clamps to the leaves in close proximity to those that had been marked. The heat tolerance test lasted for 30 min at target temperatures (43–49°C in 2 K steps for both light and dark mode). These target temperatures were determined in preceding tests and covered the range from 0 to 100% leaf tissue damage. After the heat treatment, the exposure chambers were removed and plants were exposed to the natural climatic conditions at the study site for 3 days until viability assessment was conducted.

#### Visual assessment of viability

Heat‐induced leaf injuries become visible in most plant species as clearly distinguishable discolorations due to membrane damage, loss of cell integrity and cell death. These discolorations are frequently used to assess viability wherever leaf injuries can be detected in this way (Gauslaa 1984, Buchner and Neuner 2001, 2003). For the visual assessment of heat injuries, the damaged leaf area is estimated as a percentage of the whole leaf area. In preliminary tests the visual estimation method (VEM) was validated by comparison with the visual assessment method (VAM) using an image analyses software (optimas 6.5, Optimas Corp., Seattle, WA). The results of both methods were compared but no statistical differences could be found between the VEM and the VAM (see also Buchner et al. 2013). On detached leaves that had been heat treated ex situ the VAM was used. As leaves remained attached to the plant after in situ heat treatment, it was not possible to take reliable images of the leaves because of overlapping and the risk of optical distortions, and only VEM was used.

#### Assessment of viability by chlorophyll fluorescence (F_*v*_/F_*m*_ *)*

Chlorophyll fluorescence parameters are excellent indicators of the physiological condition of the photosynthetic apparatus (for review see Goltsev et al. 2016). In vivo chlorophyll fluorescence originates entirely from the antenna complexes of PSII, which is one of the most heat susceptible parts of the photosynthetic apparatus (Larcher 2001, Schreiber and Berry 1977. *F*_v_/*F*_m_ is a frequently used parameter for the assessment of PSII, indicating the maximal photochemical efficiency of PSII, which declines with increasing stress conditions (Neuner and Buchner 1999, Dongsansuk et al. 2013, Buchner et al. 2015). The reduction in *F*_v_/*F*_m_ can also be correlated with leaf tissue damage (Larcher 2001), although not in all cases (Neuner et al. 1999, Neuner and Pramsohler 2006. *F*_v_/*F*_m_ was determined on dark adapted leaves (30 min) before and after the heat treatment using a portable chlorophyll fluorimeter (PEA MK2, Hansatech Instruments Ltd., Norfolk, UK) and was calculated by using minimum fluorescence (*F*_0_) and maximal fluorescence (*F*_m_) during a saturating red light pulse [*F*_v_/*F*_m_ = (*F*_m_ − *F*_0_)/*F*_m_]. The percentage heat damage [LT (%)] was calculated according to Buchner et al. (2013) (Eq. (1)), where (*F*_v_/*F*_m_)_T_ is the *F*_v_/*F*_m_ value of a sample after exposure to a certain treatment temperature, (*F*_v_/*F*_m_)_0%_ is the *F*_v_/*F*_m_ value of an uninjured sample exposed to a non‐harmful temperature.

(1) $$\mathrm{LT}\left[\%\right]=100\times \frac{{\left(\mathrm{Fv}/\mathrm{Fm}\right)}_{T}}{{\left(\mathrm{Fv}/\mathrm{Fm}\right)}_{0\%}}$$

### Analyses of selected antioxidants and pigments

For biochemical analysis leaves were collected after each respective heat treatment. In case of short‐term heat spell treatment leaves were harvested immediately after the 180 min exposure (150 min short‐term heat treatment, 30 min heat tolerance test) from the heat‐treated twigs. Leaves from untreated plants were collected from the same stand. Biochemical analyses were performed on leaves exposed to the highest temperature they were able to withstand without heat damage to the leaf tissue (45°C and untreated leaves). In the case of long‐term heat treatment, leaves were harvested between 17:00 and 19:00 h on day 6 directly from the three HHCs (PPFD60%, PPFD30%, PPFD12%). Untreated leaves were sampled in close proximity. The randomly harvested leaves were placed in small paper bags and immediately frozen in liquid nitrogen. The samples were transferred within half a day from liquid nitrogen to an −80°C freezer were they were stored for several weeks. Samples were then freeze dried (Lyovac GT 2, Leybold‐Heraeus, Köln, Germany) for 72 h and ground to a fine powder using a TissueLyser II (Qiagen, Düsseldorf, Germany). The powder was transferred to Eppendorf tubes (1.5 ml) and stored again in a −80°C freezer until analysis.

#### Free radical scavenging activity

To determine the total free radical scavenging activity protocols from Brand‐Williams et al. (1995) and Fukumoto and Mazza (2000) were used which are based on the turnover of the stable free radical 2,2‐diphenyl‐1‐picrylhydrazyl (DPPH). Five milligrams of freeze‐dried leaf material was transferred to a brown (light‐protected) Eppendorf tube (1.5 ml), suspended in 1 ml methanol (100%) and shaken at 4°C for 16 h. Then, the sample was centrifuged (13 000 *g*) at 4°C for 5 min, the supernatant decanted to a new Eppendorf tube and stored on ice. Twenty‐two microliter of the supernatant was placed in each well of a 96‐well microtitration plate (Bio‐Rad Laboratories, Hercules, CA), mixed with 200 μl of DPPH solution (150 μ*M* DPPH in 80% v/v ethanol) and kept in the dark. The turnover of DPPH, resulting in a decrease in absorbance, was measured with a plate reader (Multiskan EX Photometer, Thermo Fisher Scientific Inc., Waltham, MA) at 520 nm after 5, 10, 15, 20, 25, 30, 35, 60 and 90 min. To construct a calibration curve the artificial antioxidant Trolox (6‐hydroxy‐2,5,7,8‐tetramethylchroman‐2‐carbonacid) was used (500, 400, 300, 200, 100, 50 and 0 μ*M* Trolox in 100% v/v methanol). Free radical scavenging activity was expressed in Trolox equivalents (TE) normalized to dry mass.

#### Ascorbate

The total ascorbate (AA_tot_) and dehydroascorbate (DHAA) were determined by using a slightly modified protocol of Kampfenkel et al. (1995). Ten milligrams of freeze‐dried leaf powder was transferred to an Eppendorf tube (1.5 ml), suspended in 1 ml HCl [0.1 m*M* HCl + 1 m*M* EDTA (ethylenediaminetetraacetic acid) in aq. dest.], vortexed and centrifuged (15 000 *g*) at 4°C for 5 min. The supernatant was transferred to a new Eppendorf tube (1.5 ml), diluted with 0.4 *M* Sörensen buffer (pH 7.4) at a ratio of 1:3 and kept on ice. For measurement of total ascorbate (AA_tot_ = AA + DHAA) 50 μl of the diluted supernatant was mixed with 50 μl of dithiothreitol solution (5 m*M* DTT in 0.4 *M* Sörensen buffer, pH 7.4) and incubated at room temperature for 15 min. Subsequently, 25 μl of N‐ethylmaleimid solution [0.5% w/v NEM (N‐ethylmaleimid) in 2‐propanol] were added and the sample was vortexed. After adding 200 μl of working reagent (WR), consisting of WR A [4.6% w/v trichloroacetic acid, 15.3% v/v orthophosphoric acid, 0.6% FeCl_3_·6 H_2_O w/v in distilled water (aq. dest.)] and WR B (4% bipyridil in 70% ethanol) at a ratio of 11:4 (v/v), and the sample was transferred to a water bath at 42°C for 45 min.

For determination of AA 50 μl of the diluted supernatant were mixed with 50 μl of Sörensen buffer (pH 7.4) and 25 μl of NEM solution and vortexed for 5 s. Subsequently, 200 μl WR were added to the sample, followed by incubation at 42°C for 45 min. The increase in absorption due to the formation of Fe^2+^‐2,2′‐bipyridyl–complexes induced by ascorbate was measured at 520 nm using a spectrophotometer. A calibration curve was constructed for AA_tot_ and AA using an ascorbate standard (1.7, 3.3, 6.7, 13.3, 20.0 nmol AA ml^−1^). DHAA was calculated with the following equation: DHAA = AA_total_ − AA. Five technical replicates were measured for each treatment.

#### Pigment analysis

Pigments were analyzed based on the protocol of Pfeifhofer et al. (2002). Fifty milligrams of freeze‐dried leaf powder was transferred to a brown Eppendorf tube (1.5 ml) together with a spatula tip of CaCO_3_ and suspended in 500 μl of dimethylformamide (DMF). After vortexing the sample for 30 s it was kept at −21°C for at least 12 h. Afterward samples were centrifuged (20 000 *g*) at 4°C for 5 min, the supernatant was transferred to a new Eppendorf tube and stored at −21°C. These steps were repeated until the pellet was pigment‐free. In the case of *V. gaultherioides* the pellet was resuspended one time with 500 μl and two times with 250 μl DMF. Five hundred microliter of the collected supernatant were mixed with 250 μl of 50% v/v methanol and centrifuged (20 000 *g*) at 4°C for 20 min. The supernatant was used for high performance liquid chromatography (HPLC) analysis of pigments (Agilent ChemStation 1100, Agilent Technologies, Santa Clara, CA). Pigments were separated on a LiChroSpher C18 column (Phenomenex Inc., Torrance, CA) at a flow rate of 1 ml min^−1^. Pigments were identified by retention time and absorption spectra using a diode array detector. Calibration curves for quantification were constructed using external standards (chlorophyll a: Sigma Aldrich, St. Louis, MO; ß‐carotene: Calbiochem, Darmstadt, Germany; antheraxanthin and violaxanthin: DHI, Hørsholm, Denmark; zeaxanthin and lutein: Carl Roth, Karlsruhe, Germany). Chlorophyll b and neoxanthin were collected using an Agilent 1200 Series fraction collector (Waldbronn, Germany). Absorption coefficients, as described by Pfeifhofer et al. (2002), were used to calculate pigment concentrations before being used as external standards. Five technical replicates were measured for each treatment in each experiment.

### Transmission electron microscopy

To detect alterations in the ultrastructure of cells, especially chloroplasts and thylakoid membranes, due to long‐term heat treatments under different light conditions (PPFD100%, PPFD60% and PPFD12%), leaves were harvested and prepared for transmission electron microscopy (TEM) at day 6 of the treatment (the same time as samples were taken for pigment analysis). Leaf samples were treated as described by Holzinger et al. (2007), cut into small (2 × 3 mm) pieces, vented in a vacuum and then fixed in glutaraldehyde solution (2.5% w/v in 50 m*M* cacodylat buffer, pH 7) for 1.5 h, in 1% OsO_4_ solution overnight, washed several times with aq. dest., and then stored in cacodylate buffer at 4°C for several weeks. Subsequently, leaves were dehydrated in increasing ethanol concentrations and embedded in low viscosity embedding media (Spurr Low Viscosity Embedding Kit, Serva, Heidelberg, Germany). Ultrathin sections were poststained and analyzed using a TEM (Libra 120, Carl Zeiss, Oberkochen, Germany) at 80 kV and pictures were taken with a CCD camera (Slow Scan, ProScan, Lagerlechfeld, Germany).

### Statistics

Statistical analyses were conducted using SPSS software (IBM SPSS‐Statistics 21, New York, NY). Differences between mean values were tested by one‐way anova and Duncan's multiple range test (*P* < 0.05) or by the Student's *t*‐test (*P* < 0.05) after testing for normality by the Kolmogorov–Smirnov test. If homogeneity of variances was not given, Tamhane T2‐test was used.

## Results

### Microclimate

**Figure 1:**
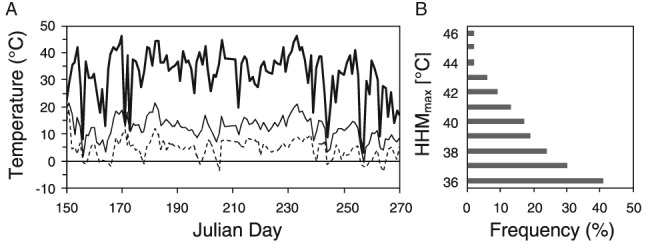
Leaf temperatures of Vaccinium gaultherioides. HHM of leaf temperatures were recorded on Mt. Patscherkofel (1950 m a.s.l.) during the summer of 2012. Thermocouple sensors were attached to the lower leaf surfaces (n = 8) and data were recorded at 1 min intervals. (A) The dashed line shows daily minimum HHM, the thin solid line shows daily mean HHM and the thick solid line shows daily maximum HHM. (B) Percentage of days (frequency) for which the maximum HHM values (HHM_max_) were exceeded at least once a day.

The summer of 2012 was the third warmest summer in Austria since the beginning of systematic climate records (ZAMG 2012). Increased air temperatures also had a significant impact on leaf temperatures. On several days leaf temperature maxima exceeded 44°C (HHM temperature; June 18: 46.1°C, June 30: 44.2°C, July 01: 44.3°C, July 24: 44.8°C) and on August 20 a summer maximum of 46.4°C was recorded (Fig. 1A). On 17% of observation days, daily leaf temperatures exceeded 40°C (HHM, Fig. 1B). Heat waves in June (June 18: 46.1°C) and August (August 20: 46.4°C) were followed by a drastic drop in maximum HHM to 14.5°C (June 19) and 8.7°C (August 21), respectively.

### Effects of short‐term heat spell treatment under low solar irradiation, and in darkness

#### Photosynthetic efficiency and heat tolerance after short‐term heat spell treatment

An in situ short‐term heat spell treatment (30 min at 30°C and in darkness; 120 min heating to target temperatures, and a final 30 min exposure to 43, 45, 47 or 49°C), either under low solar irradiation (light mode; maximum PPFD 110 and 250 μmol m^−2^ s^−1^ during linear heating and at exposure temperatures, respectively) or in full darkness (dark mode), followed in both cases by recovery under natural environmental conditions, resulted in a reduction in potential photosynthetic efficiency (*F*_v_/*F*_m_). However, in the dark (D) reduction in *F*_v_/*F*_m_ was always more pronounced than in the light (L).

**Figure 2:**
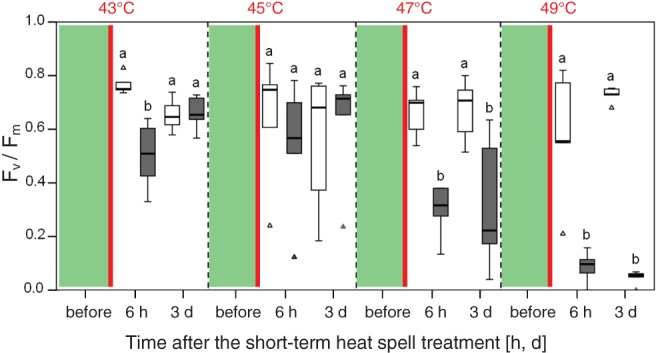
Effects of short‐term heat spell treatments on photosynthetic efficiency (F_v_/F_m_) of Vaccinium gaultherioides. Using the HTTS, plants were allowed to equilibrate for 30 min at 30°C, then heat spells were applied by increasing the temperature over 120 min from 30°C to 43, 45, 47 or 49°C, followed by holding these temperatures for 30 min, either under low light (white boxplots) or in darkness (gray boxplots). Heat treatments (red vertical lines) were started before sunrise, and F_v_/F_m_ of the same leaves (n = 5) was measured the day before the heat treatment (green boxes), 6 h and 3 days after the heat treatment (3). Different exposure temperatures are separated by dashed vertical lines. Significant differences in F_v_/F_m_ between light and dark on the same day are indicated by different letters (Student's t‐test, P < 0.05). Box plots show medians, 25 and 75% percentiles, and whiskers extend to a maximum of 1.5 times the box height. Triangles represent outliers.

**Figure 3:**
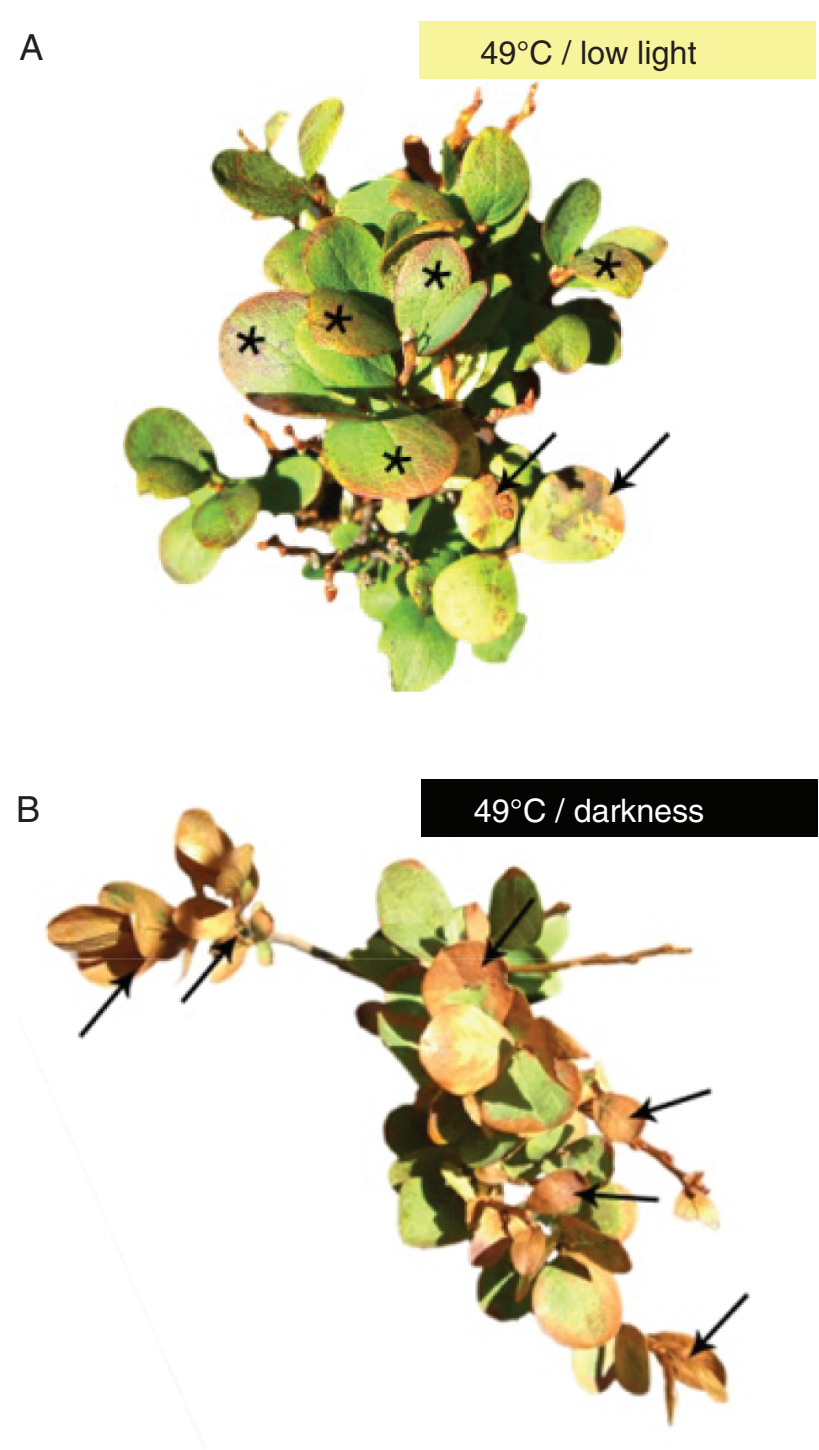
Tissue damage after short‐term heat spell treatment. Leaves of Vaccinium gaultherioides were exposed to a heat spell in situ by equilibrating the plant for 30 min at 30°C, followed by linearly increasing the temperature from 30 to 49°C within 120 min, then holding the final temperature for 30 min (A) under low light and (B) in darkness. Photographs were taken after 3 days of recovery under natural environmental conditions. Arrows denote necrotic leaf tissue and (undamaged) leaf colorations are marked with stars.

In the light even after exposure to the highest treatment temperature (49°C), 6 h after the heat spell treatment *F*_v_/*F*_m_ was only slightly reduced by 30.4% but in the dark *F*_v_/*F*_m_ was reduced by 89.5% (Fig. 2). Similarly, recovery of *F*_v_/*F*_m_ after 3 days under the natural ambient field conditions was less in leaves that had been heat‐treated in the dark. Leaves exposed to 43 and 45°C were able to recover in both, L and D, but leaves exposed in the dark to 47°C showed only a slight, not significant, recovery of *F*_v_/*F*_m_ (from 0.30 ± 0.1 after 6 h to 0.32 ± 0.25 at day 3), and no recovery at all when exposed to 49°C (Fig. 2). Generally, heat damage to leaves occurred only in the two highest heat spell treatments (47 and 49°C). Again, after 3 days leaves exposed in the dark showed 15% (47°C) and 60% (49°C) heat damage, and leaves exposed in the light showed only 5% damage when exposed to 47 or 49°C (Fig. 3). The LT_50 *F*v/*F*m_ (±sd) showed significant differences between leaves exposed in the light (49.8°C ± 0.5) and leaves exposed in the dark (46.1°C ± 0.9). LT_50 visual_ was >49.0°C in the light but lower in the dark (48.5°C ± 0.0).

#### Photosynthetic pigments

**Figure 4:**
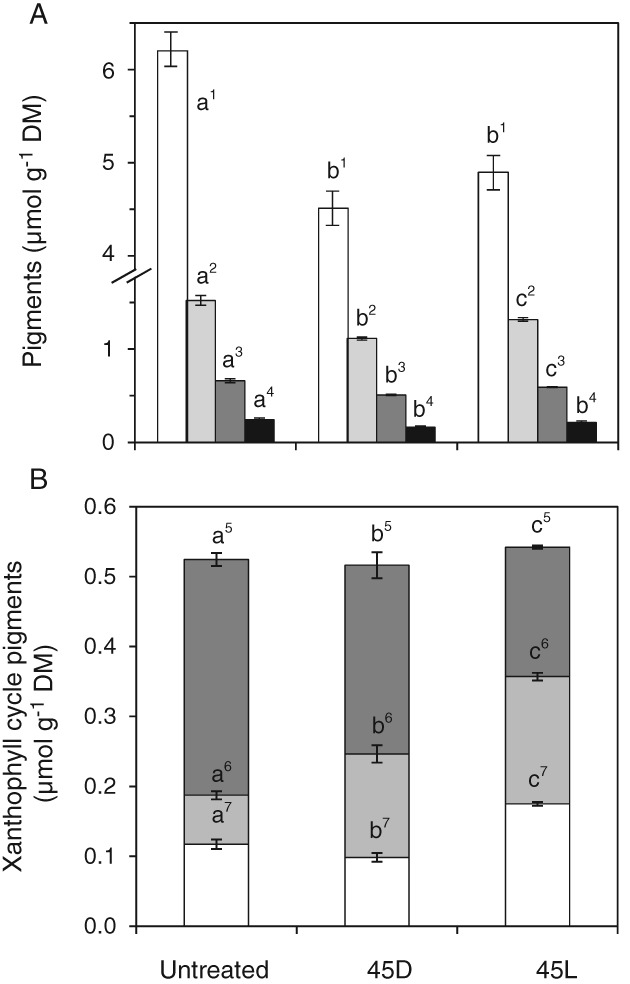
Pigment composition in response to short‐term heat spell treatment. Untreated leaves of Vaccinium gaultherioides and leaves exposed in situ to a sublethal short‐term heat spell (30 min at 30°C; 120 min heating to 45°C at 7.5 K h^−1^, followed by holding 45°C for 30 min) under low light (45L) and in the dark (45D). In (A) bars show chl a (white), chl b (light gray), lutein (dark gray) and neoxanthin (black). In (B) changes in xanthophyll cycle pigments are shown: violaxanthin (dark gray), antheraxanthin (light gray) and zeaxanthin (white). Different letters show significant differences between treatments for each pigment (one‐way anova, Duncan's test, P < 0.05). DM, dry mass. Data are means ± sd (n = 5).

In leaves exposed to 45°C, the pigment concentrations (chlorophyll a and b, lutein, neoxanthin) were significantly (*P* < 0.05) reduced in the light and in the dark compared to untreated plants (Fig. 4A). The xanthophyll cycle pigments violaxanthin (V), antheraxanthin (A) and zeaxanthin (Z) were also significantly (*P* < 0.05) affected by both treatments (Fig. 4B). The effect was more pronounced in the light where more violaxanthin was de‐epoxidized to zeaxanthin. The higher de‐epoxidation status in the light resulted mainly from an increase in zeaxanthin, whereas leaves treated in the dark showed no increase in zeaxanthin compared to untreated plants. The ratio Z/(V + A + Z) was 22.3% in untreated leaves, 19.1% in the dark and 31.5% in the light. Antheraxanthin amounts were increased in both, the light and the dark, compared to untreated plants. The ratio A/(V + A + Z) was 13.4% in untreated plants, 28.6% in the dark and 34.0% in the light. In summary, leaves subjected to heat stress in the light contained the highest antheraxanthin and zeaxanthin amounts and had the highest heat tolerance.

#### Free radical scavenging activity and ascorbate

**Figure 5:**
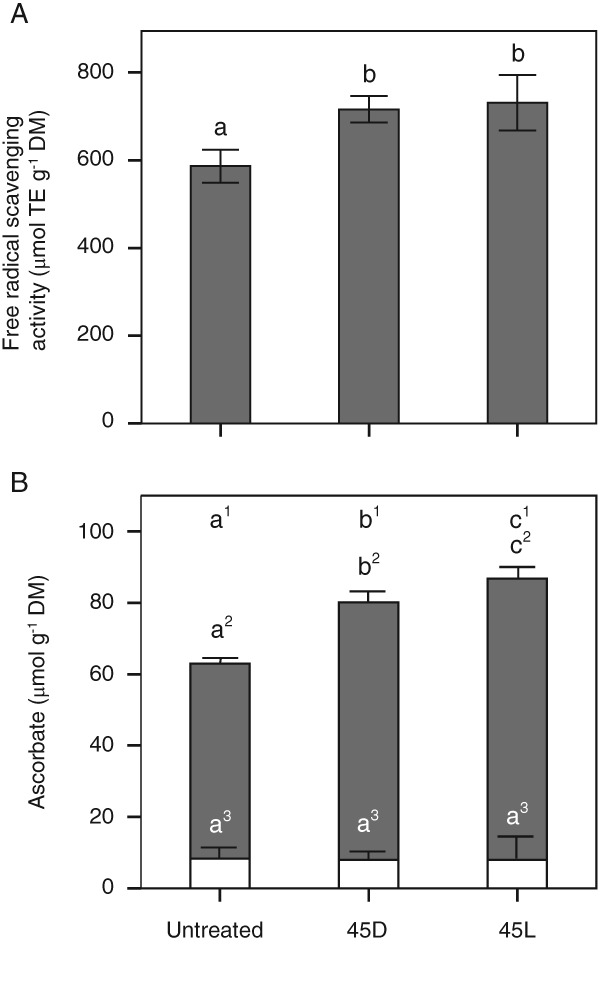
Free radical scavenging activity and ascorbate concentration in response to short‐term heat spell treatment. Untreated leaves of Vaccinium gaultherioides and leaves exposed in situ to a sublethal short‐term heat spell (30 min at 30°C; 120 min heating to 45°C at 7.5 K h^−1^, followed by holding 45°C for 30 min) under low light (45L) and in the dark (45D). (A) Free radical scavenging activity expressed as TE per dry mass (DM), and (B) ascorbate (gray bars) and dehydro‐ascorbate concentration (white bars). Different letters indicate significant differences between treatments, letters at the top of B correspond to total ascorbate concentration (one‐way anova, Duncan's test, P < 0.05). Data are means ± sd (n = 5).

The free radical scavenging activity significantly (*P* < 0.05) increased in leaves exposed to 45°C in the light and the dark compared to untreated plants (Fig. 5A). Also the total amount of ascorbate was significantly (*P* < 0.05) higher in heat‐treated plants in which the increase was significantly higher in the light than in the dark (Fig. 5B). The increase in ascorbate was due to an increase in AA, while DHAA levels remained unchanged by the heat and light treatments.

### Effects of long‐term heat wave treatment under different solar irradiation levels

#### Solar irradiation levels and leaf temperatures

Using the HHCs it was possible to simulate long‐term heat waves (7 days) under three solar irradiation levels: untreated plants (PPFD100%) received a mean PPFD during daytime of 985 μmol m^−2^ s^−1^ followed by PPFD60% (585 μmol photons m^−2^ s^−1^), PPFD30% (276 μmol m^−2^ s^−1^) and PPFD12% (115 μmol photons m^−2^ s^−1^). During daytime a mean leaf temperature of 30°C could be nicely simulated, ranging between 30.0 and 30.6°C in all three light intensity scenarios (Table 1). Compared to the mean leaf temperature of untreated plants (20.3°C) the leaf temperatures in the HHCs were increased by 10 K (Table 1). Due to solar irradiation input, different leaf angles and orientation absolute maximum temperatures of single leaves inside the HHCs ranged between 43.8 and 44.9°C but were comparable to leaves of untreated plants from outside (44.3°C) (Table. 1). Such peak temperatures occurred only in single leaves for a maximum time span of about 10–15 min and did not cause any heat damage. Peak temperatures ≥40°C were detected at relative frequencies of 1.0% (untreated, PPFD100%), 2.4% (PPFD60%), 2.9% (PPFD30%) and 0.1% (PPFD12%).

**Table 1:** Exposure of Vaccinium gaultherioides to a long‐term heat wave. Heat‐treatment took place for 7 days under different solar irradiation conditions (PPFD) inside light transmissive HHCs. During daytime, mean leaf temperatures were set to 30°C (nighttime: 15°C). Mean (±sd) and short‐term (up to max. 15 min) occurring maximum (max) and minimum (min) leaf temperatures inside the HHCs and on untreated shrubs are given.

	PPFD (%)	100% (untreated)	60%	30%	12%
Leaf temperature [°C]					
Day	Mean ± sd	20.3 ± 8.7	30.6 ± 5.3	30.5 ± 5.5	30.0 ± 6.3
	Max	44.3	43.8	44.9	43.6
Night	Mean ± sd	10.1 ± 2.7	15.4 ± 0.9	15.1 ± 1.1	14.0 ± 2.6
	Min	2.3	12.3	9.0	5.7

#### Photosynthetic efficiency and heat tolerance during and after long‐term heat wave treatment

**Figure 6:**
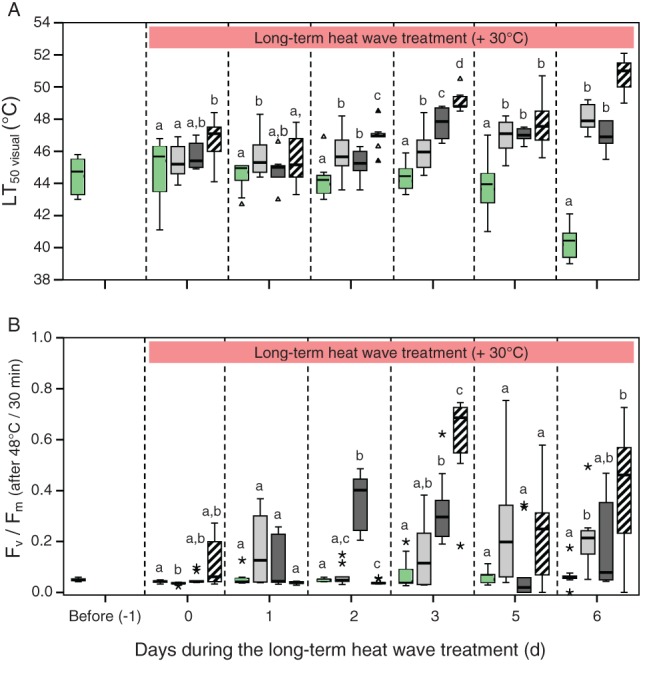
Dynamics of heat hardening in leaves of Vaccinium gaultherioides during exposure to an in situ long‐term heat wave treatment under different solar irradiation intensities. In special HHC's plants were exposed to 30°C (mean leaf temperature) during day and 15°C during night for 7 days under three different mean light intensities (PPFD): 585 μmol photons m^−2^ s^−1^ (PPFD60%, light gray), 276 μmol photons m^−2^ s^−1^ (PPFD30%, dark gray), 115 μmol photons m^−2^ s^−1^ (PPFD12%, hatched). Untreated leaves (green), received a mean light intensity of 985 photons m^−2^ s^−1^ (100%). (A) Heat tolerance of leaf tissue (LT_50 visual_) and (B) potential quantum efficiency of PSII (F_v_/F_m_) was tested daily (except day 4) on detached leaves. F_v_/F_m_‐values are shown for leaves exposed to 48°C during the heat tolerance test only. Significant differences in LT_50 visual_ or F_v_/F_m_ between the four treatments within 1 day are indicated by different letters (one‐way anova, Duncan's or Tamhane T2‐test, P < 0.05). Box plots show medians, 25 and 75% percentiles, and whiskers extend to a maximum of 1.5 times the box‐height. Triangles or asterisks represent outliers.

In untreated plants (PPFD100%) no significant (*P* < 0.05) changes in heat tolerance (LT_50_) were observed until day 5, but LT_50_ declined on day 6. In contrast, heat hardening was triggered by all long‐term heat wave treatments (PPFD60%, PPFD30%, PPFD12%), resulting in a significant (*P* < 0.05) increase in heat tolerance (Fig. 6A). The highest overall heat hardening was observed under low light (PPFD12%). Already after 6 h of heat treatment leaves of PPFD12% showed a significantly increased LT_50 visual_ and on 4 of 6 days (days 0, 2, 3, 6) the LT_50 visual_ of PPFD12% tended to be higher or was significantly (*P* < 0.05) higher than that of the other three long‐term heat wave treatments. On day 6 the heat tolerance reached the highest values, and PPFD12% had the highest LT_50 visual_ of 50.9°C ± 1.0 followed by PPFD60% (48.1°C ± 0.8) and PPFD30% (46.9°C ± 0.8). The lowest LT_50visual_ values were determined for untreated plants (40.3°C ± 1.1).

In addition, in heat‐treated plants (PPFD60%, PPFD30% and PPFD12%) heat tolerance of PSII was significantly greater. When leaves were exposed to 48°C for 30 min during the heat tolerance test, PSII of untreated leaves was completely damaged on almost all days (Fig. 6B). By contrast, heat‐treated leaves showed only partially reduced *F*_v_/*F*_m_ throughout the experiment (PPFD60% and PPFD30%). Additionally, in PPFD12% *F*_v_/*F*_m_ was highest on 3 of 7 days (days 0, 3, 6). Thus, also LT_50 *F*v/*F*m_ increased in heat‐treated plants during the long‐term heat wave treatment, and on day 6 LT_50 *F*v/*F*m_ followed the same order as LT_50 visual_, and were only marginally lower (PPFD12%: 48.0°C ± 1.2; PPFD60%: 46.6°C ± 0.7; PPFD30%: 46.3°C ± 0.7; untreated plants: 42.1°C ± 0.7).

**Figure 7:**
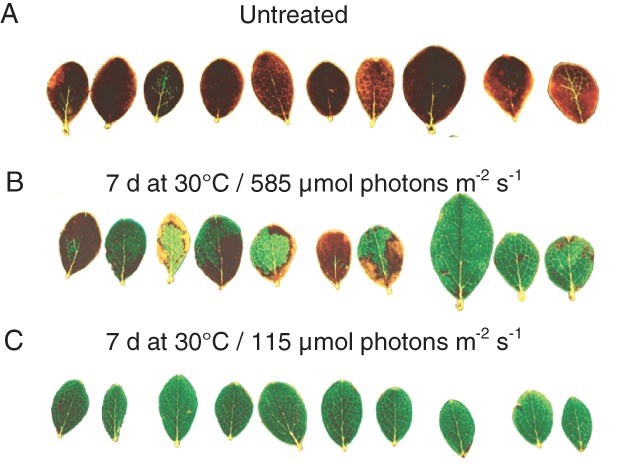
Heat tolerance after long‐term heat wave treatment. Leaves of Vaccinium gaultherioides after a 30 min exposure to 48°C using MHCs. Leaves from untreated plants (A) are compared to leaves that had been exposed in situ to a long‐term heat wave (7 days; 30°C/15°C day/night) either under (B) 585 μmol photons m^−2^ s^−1^ (PPFD60%) or (C) 115 μmol photons m^−2^ s^−1^ (PPFD12%). Heat damaged leaf tissue can be seen as necrotic, brownish discolorations.

Differences in heat tolerance were also clearly seen in PPFD60% and PPFD12% leaves exposed during the heat tolerance test to 48°C for 30 min on day 6. After heat exposure at 48°C PPFD12% leaves showed no tissue damage, PPFD60% leaves showed either no tissue damage, were only partly damaged or were killed, whereas all untreated leaves (PPFD100%) died (Fig. 7). In summary, long‐term heat wave treatment induced heat hardening, which was greatest at the lowest light intensity of 115 μmol m^−2^ s^−1^ whereas no heat hardening was observed in untreated plants.

#### Photosynthetic pigments

**Figure 8:**
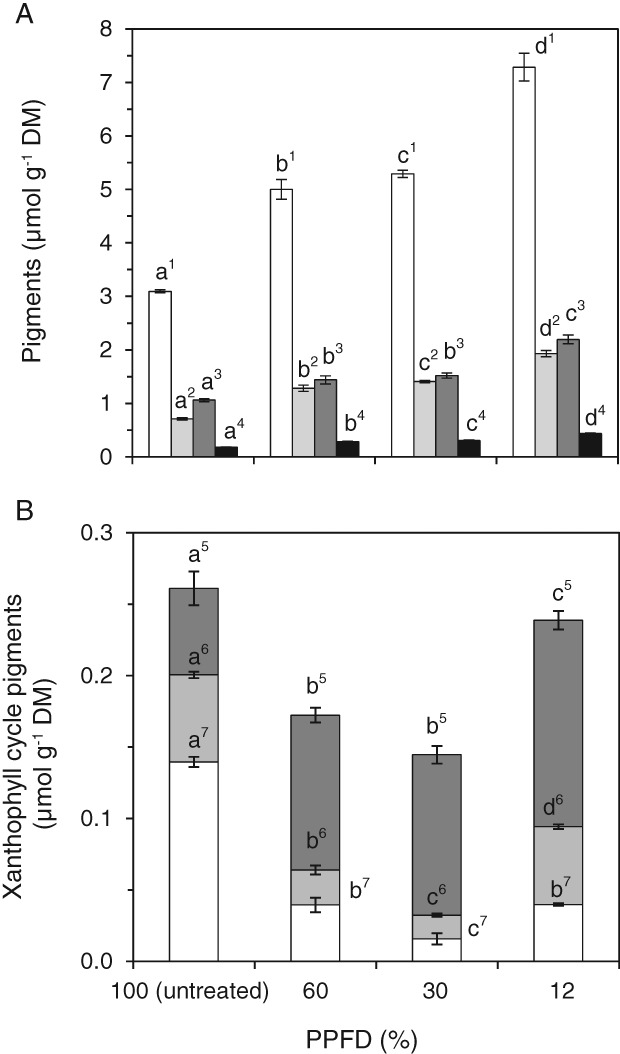
Pigment composition in response to a long‐term heat wave. Untreated leaves (PPFD100%) of Vaccinium gaultherioides and leaves exposed in situ to a long‐term heat wave for 7 days with leaf temperatures of 30°C during day and 15°C during night and mean light intensities (PPFD) ranging from 585 μmol photons m^−2^ s^−1^ (PPFD60% %) to 276 μmol photons m^−2^ s^−1^ (PPFD30% %) and 115 μmol photons m^−2^ s^−1^ (PPFD12% %). In (A) bars show chl a (white), chl b (light gray), lutein (dark gray) and neoxanthin (black). In (B) changes in xanthophyll cycle pigments are shown: violaxanthin (dark gray), antheraxanthin (light gray) and zeaxanthin (white). Different letters show significant differences between treatments for each pigment (one‐way anova, Duncan's test, P < 0.05). Data are means ± sd (n = 5). DM, dry mass.

The amount of the pigments chl a, chl b, lutein and neoxanthin significantly (*P* < 0.05) increased with decreasing light intensity (Fig. 8A). Compared to untreated plants, PPFD12% leaves showed a 2.5‐fold increase in neoxanthin, and lutein, chl a and chl b increased 2.07‐, 2.4‐ and 2.7‐fold, respectively. The chl a to chl b ratio was significantly (*P* < 0.05) lowered from 4.4 (untreated) to 3.9 (PPFD60%) and 3.8 (PPFD30%, PPFD12%). Xanthophyll cycle pigments were also significantly (*P* < 0.05) affected by the long‐term heat treatment (Fig. 8B). The total amount of xanthophyll cycle pigments (V + A + Z) was highest in untreated plants (PPFD100%) and in PPFD12% leaves, but about one third lower in PPFD60% and PPFD30% leaves. The highest amounts of antheraxanthin and zeaxanthin were found in untreated plants whereas in the heat‐treated plants antheraxanthin and zeaxanthin were epoxidized to a high degree to violaxanthin (Fig. 8B).

#### Free radical scavenging activity and ascorbate

**Figure 9:**
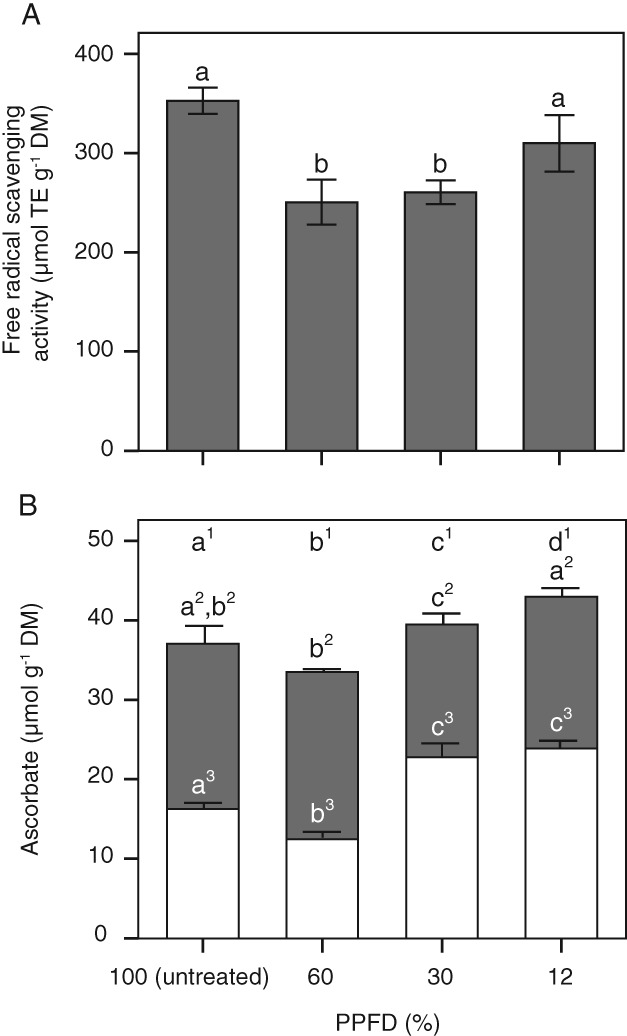
Free radical scavenging activity and ascorbate concentration in response to a long‐term heat wave. Untreated leaves of Vaccinium gaultherioides (PPFD100%) and leaves exposed in situ to a long‐term heat wave for 7 days with leaf temperatures of 30°C during day and 15°C during night and mean light intensities (PPFD) ranging from 585 μmol photons m^−2^ s^−1^ (PPFD60%) to 276 μmol photons m^−2^ s^−1^ (PPFD30%) and 115 μmol photons m^−2^ s^−1^ (PPFD12%). (A) Free radical scavenging activity expressed as Trolox equivalents per dry mass (DM), and (B) ascorbate (gray bars) and dehydro‐ascorbate concentration (white bars). Different letters show significant differences between treatments (one‐way anova, Duncan's test, P < 0.05). Data are means ± sd (n = 5).

Free radical scavenging activity was highest in untreated plants (PPFD100%). In contrast, in PPFD60% and PPFD30%, a significant (*P* < 0.05) decline in free radical scavenging activity by about 30% was found compared to PPFD100%, whereas in PPFD12% leaves it only declined by 12% (Fig. 9A). The highest amounts of total ascorbate were found in PPFD30% and PPFD12% leaves that also showed significantly (*P* < 0.05) higher levels of DHAA (Fig. 9B).

#### Cellular ultrastructure: TEM

**Figure 10:**
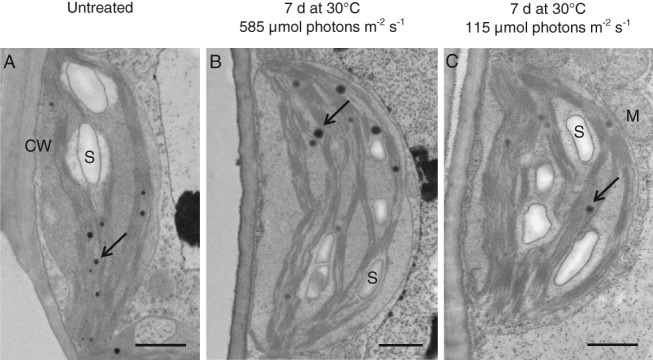
Effects of a long‐term heat wave on chloroplast ultrastructure. TEM images of chloroplast ultrastructure of (A) untreated leaves of Vaccinium gaultherioides and leaves exposed in situ to a long‐term heat wave for 7 days with mean leaf temperatures of 30°C during day and 15°C during night and mean light intensities of (B) 585 μmol photons m^−2^ s^−1^ (PPFD60%) and (C) 115 μmol photons m^−2^ s^−1^ (PPFD12%). CW: cell wall; M: mitochondria; S: starch. Black arrow: plastoglobuli. Scale bars: 1 μm.

The TEM images obtained from cross sections of leaves collected after 7 days of long‐term heat treatments (PPFD60% and PPFD12%) and from untreated plants (PPFD100%) showed that neither chloroplast ultrastructure, nor thylakoid stacking nor the establishment of starch grains were noticeably affected by the heat treatments (Fig. 10).

## Discussion

### Effects of solar irradiation during short‐term heat spell treatment on heat tolerance of leaf tissue and PSII

The short‐term heat spell treatment mimicked naturally occurring short‐term heat spells such as those that can occur in the alpine zone during midday hours. Application of low light during the short‐term heat spell treatment had a significant protective function on PSII and the leaf tissue. *F*_v_/*F*_m_ only declined slightly when low light was applied during short‐term heat spells whereas leaves exposed to heat in darkness showed a drastic decline in *F*_v_/*F*_m_ after the recovery phase, often in conjunction with leaf tissue damage. This damage became irreversible after exposure to 49°C (Fig. 2). The calculated heat tolerance (LT_50 *F*v/*F*m_) was significantly (*P* < 0.05) higher by 3.7 K in leaves of *V. gaultherioides* that had been heat treated under low light than in leaves that had been treated in darkness. A protective function of low light during a short‐term heat spell treatment was also confirmed after plant exposure to 47 and 49°C in the light, causing only 5% damage, whereas these temperature led to significant leaf damage in the dark. This protective role of light on the heat stability of PSII has already been demonstrated in laboratory‐based experiments (Schreiber and Berry 1977, Havaux et al. 1991), but also in a recent field study on other alpine plant species (*R. glacialis*, *Rhododendron ferrugineum* and *Senecio incanus*; Buchner et al. 2015). Earlier reports indicate that low light intensities (approximately 200/380 μmol photons m^−2^ s^−1^) can have a protective effect on the heat stability of PSII (Weis 1982, Havaux et al. 1991). During our short‐term heat spell treatments leaves received a comparable light intensity with a maximum of 250 μmol photons m^−2^ s^−1^.

The mechanisms responsible for increased heat stability of PSII and for leaf tissue protection under light exposure are still not fully understood. The observed protective effect of low light during short‐term heat stress on PSII and leaf tissue could be explained by a decrease in Rubisco activity due to misprotonation during heat stress, producing the inactive form of the enzyme, and by a reduction of the activity of the heat sensitive rubisco activase (Salvucci 2004, Salvucci and Crafts‐Brandner 2004, Kim 2005). The regulation of rubisco activase is principally linked to light via the ferredoxin‐thioredoxin system. Rubisco activase activity is also influenced by ATP to ADP ratio (Portis et al. 2007). Low ATP levels in darkness would suggest that rubisco activase activity was reduced in leaves that were heat exposed in darkness. Additionally, leaves that were heat treated in darkness were exposed to natural light conditions after the heat stress. After heat stress the lowered rubisco activase activity could have led to an impairment of the Calvin cycle leading to an electron tailback, an over reduction of the photosystem, therefore causing damage to PSII and leaf tissue. Immediately after the heat treatment leaves of *V. gaultherioides* were exposed only to moderate light intensities (on average 400 μmol photons m^−2^ s^−1^, maximum 800 μmol photons m^−2^ s^−1^). However, after midday PPFD increased up to ca. 2000 μmol photons m^−2^ s^−1^. It is well known that light intensities in high alpine regions can rise from low levels (<50 μmol photons m^−2^ s^−1^) under a cloudy sky to >2000 μmol photons m^−2^ s^−1^ in full sunlight within a very short space of time. The photosynthetic apparatus in alpine plants has evolved to withstand such rapid changes in light intensity. However, it cannot be completely ruled out that the sudden change in light intensities (from darkness to low/moderate sunlight) was co‐responsible to some extent for the higher damage of PSII and leaf tissue in leaves that were heat treated in darkness.

In sunlight photosynthesis and many other metabolic pathways are operative. The accumulation of HSP and especially sHSP, which was found to increase in response to combined heat and light stress (Barua and Heckathorn 2006), could be among the factors responsible for the higher heat tolerance of PSII and leaf tissue. Especially, sHSP seem to protect PSII, the electron transport chain and the water splitting complex during heat shocks (Heckathorn 1998, Preczewski et al. 2000). In addition, increased sugar concentration and osmotic potential under light, could have protective effects on PSII, the Calvin cycle and the electron transport chain (Hüve et al. 2006). The plastoquinol terminal oxidase (PTOX) is a further possibility for plants to transfer electrons from plastoquinol onto O_2_ to form H_2_O. High levels of PTOX have been found in several alpine plant species (Streb et al. 2005, Laureau et al. 2013) and a protective role during combined heat and light stress events has been suggested (McDonald et al. 2011).

### Effects of short‐term heat spell treatment on leaf pigments, free radical scavenging activity and ascorbate

Leaves exposed to 45°C showed a significant (*P* < 0.05) decrease in leaf pigments, except for xanthophyll cycle pigments, in both the light and the dark. In an earlier study on *R. glacialis* exposed to 38°C no such decrease in the concentration of leaf pigments was detected (Streb et al. 2003). However, at 45°C, close to the temperature at which first leaf tissue damage was detected, pigments could have been partly destroyed or de novo pigment synthesis could have failed. Weis (1982) and Havaux et al. (1991) showed in laboratory experiments that light‐induced acidification of the thylakoid lumen increased the heat stability of thylakoid membranes and PSII. This acidification activates the xanthophyll cycle (Demmig‐Adams 2003) which is even more active when high solar irradiance is accompanied by higher temperatures (Streb et al. 2003, Yin et al. 2010, Dongsansuk et al. 2013). This induced formation of zeaxanthin contributes to non‐photochemical quenching (NPQ) and also helps protect thylakoid membranes from oxidation (Havaux 2004, Jahns and Holzwarth 2012) and decreases membrane fluidity (Havaux 1998). The increased levels of zeaxanthin found in leaves that had been heat‐treated in the light could have been partially responsible for the higher heat tolerance of PSII and leaf tissue. Interestingly, the xanthophyll cycle was not only activated in the light but also in darkness, as shown by the higher amounts of antheraxanthin in leaves that had been heat‐treated in darkness compared to untreated leaves. An activation of the xanthophyll cycle in darkness was also detected in other species including in desiccation tolerant, taxonomically distant, plants including algae, mosses and angiosperms upon desiccation in the dark (Fernández‐Marín et al. 2011) and in alpine plant species, such as *R. glacialis* and *S. incanus*, exposed to high temperatures (38 to 44°C) in darkness (Streb et al. 2003, Buchner et al. 2015).

Antioxidants play a pivotal role in surviving heat stress events during which levels of ROS are known to increase (Allakhverdiev et al. 2008). Free radical scavenging activity increased upon heat exposure in the light and in the dark, indicating an increase in production of antioxidants. This increase was confirmed for ascorbate, with a significantly (*P* < 0.05) higher concentration after heat treatment than in untreated plants. The increase was higher in the light than in the dark. An increase in ascorbate and glutathione was also found in leaves of *O. sativa* when exposed to 35°C in the light, whereas no increase was found when leaves were exposed to heat in darkness (Yin et al. 2010). Yin et al. (2010) also found increased enzyme activity of superoxide dismutase and glutathione‐reductase in rice leaves when exposed to 35°C and light, whereas no increase was found when leaves were exposed to heat in darkness. Both enzymes are involved in ROS scavenging. An increase in enzyme activity during heat stress, when applied in the light, could confer additional advantage in surviving a short‐term heat spell by preventing damage by ROS. In summary, increased free radical scavenging activity, increased ascorbate levels and enhanced xanthophyll cycle activity (Fig. 4) were found in plants without visible leaf damage and in which PSII activity was not significantly affected by short‐term heat exposure to 45°C (Fig. 2), suggesting a significant role in the successful management of heat stress. However, our ascorbate data do not allow for more detailed interpretation, because it is unclear whether differences between the treatments (light vs dark) have been caused by enhanced synthesis or reduced demand. If mean PPFD during the short‐term heat spell treatment had been higher, the influence of light vs darkness may have become more evident.

### Effects of solar irradiation intensity during long‐term heat wave treatment on cellular ultrastructure and heat tolerance of leaf tissue and PSII

The long‐term heat wave treatments were designed to simulate possible future heat waves lasting for several days as predicted by IPCC (2013), allowing heat hardening but without killing the plants. No differences in the cellular and chloroplast ultrastructure were observed between untreated leaves and heat treated PPFD60% and PPFD12% leaves, and no disruption of the grana thylakoids was detected as reported by other authors (Smillie et al. 1978, *H. vulgare*: 31, 32°C; Larcher et al. 1997, *R. glacialis*: >42°C; Vani et al. 2001, *O. sativa*: >35°C). During stress conditions HSP and sHSP can form so‐called heat shock granules in which proteins and housekeeping mRNA are protected from degradation (Dylewski et al. 1991, Forreiter et al. 1997, Kirschner et al. 2000), providing an advantage in surviving heat stress (Miroshnichenko et al. 2005). Neither in untreated leaves nor in long‐term heat‐treated leaves were such heat shock granules detected. However, the plants were able to produce sufficient photosynthate for the synthesis of transitory starch even under low light (Fig. 10). This is in line with data from M. Stoll (personal communication) revealing that, in untreated *V. gaultherioides* leaves, CO_2_‐assimilation rates at low light (PPFD 100 μmol photons m^−2^ s^−1^) still reached ca. 30% compared to full sunlight (PPFD 2000 μmol photons m^−2^ s^−1^). However, it is also conceivable that reduced degradation of starch granules, which is catalyzed by several enzymes might have been involved to some extent (reviewed by Orzechowski 2008). As leaf samples for the TEM study were taken at the late afternoon (and not before sunrise), our data neither exclude nor confirm the presence of reduced starch degradation during long‐term heat wave treatment under low light.

Long‐term heat wave treatment of *V. gaultherioides* plants triggered heat hardening. LT_50 visual_ increased by between 5.9 and 10.6 K, depending on irradiation intensity. Under low light (115 μmol photons m^−2^ s^−1^) heat hardening was greatest and led to an LT_50 visual_ value of 50.9°C, compared to an LT_50 visual_ of only 40.3°C in untreated plants. Higher light intensities (276 and 585 μmol photons m^−2^ s^−1^) led to less pronounced heat hardening with LT_50 visual_ values of 46.9 and 48.1°C, respectively. In summary, the presence of low light during both the short‐term heat spell treatment and the long‐term heat wave treatment supported the ability to survive critically high leaf temperatures, compared to darkness or higher light intensities, respectively (Figs 3, 6 and 7).

Information regarding the effects of light during exposure to long‐term heat treatment on heat hardening is scarce. Maier (1971) found increased LT_50_ values when plants were exposed to high temperatures under high light as compared to low light exposure, but this could have been an effect of higher leaf temperatures under high light, which was not measured. The underlying molecular mechanisms of heat hardening during a long‐term heat wave under low light conditions are not clearly understood. Higher cellular osmotic potentials are unlikely to explain the highest heat hardening in PPFD12%, as higher osmotic potentials are normally found in plants exposed to higher light intensities (Hüve et al. 2006). Higher concentrations of HSP and sHSP could be important factors; however, the production of HSP and sHSP is also higher when the heat exposure takes place in the light (Sun et al. 2002, Wang et al. 2004). In addition, PTOX is unlikely to be responsible for the increased heat hardening in PPFD12%, as its protective function seems to be strongest when heat and light stress are combined (McDonald et al. 2011). Modification of thylakoid membrane composition, such as replacement of unsaturated fatty acids with more saturated ones, has been suggested to be important for plants to acclimate to higher temperatures (Pearcy 1978, Raison et al. 1982, Hugly et al. 1989). Given that the heat stress of the treatments in PPFD60%, PPFD30% and PPFD12% was identical it is unlikely (although cannot be ruled out) that PPFD12% leaves had different thylakoid membrane composition compared to the other two treatments.

### Effects of long‐term heat wave treatment on leaf pigments, free radical scavenging activity and ascorbate level

Low light intensities during long‐term exposure to elevated temperatures in PPFD60%, PPFD30% and PPFD12% resulted in changes in pigment concentrations typically observed in response to low light conditions, including increased amounts of chlorophylls, lutein and neoxanthin, and a lower chl a to chl b ratio with decreasing light intensity (Lichtenthaler et al. 1981, Sarijeva et al. 2007). The decrease in chl a to chl b ratio could be due to an increased number of light‐harvesting complexes (LHCII) per PSII reaction center with decreasing light intensity (Leong and Anderson 1984). LHC complexes play an important role in the adhesion between thylakoid membranes in grana stacks (Santarius and Weis 1988). Therefore, in the context with the long‐term heat wave treatment the observed increase in pigments, indicative of changes to LHCII, could have a stabilizing effect on the thylakoid membranes, protecting them from heat damage.

Zeaxanthin, which has been shown to be involved in stabilizing thylakoid membranes during short‐term heat events, is unlikely to be directly involved in long‐term heat hardening because the highest amounts of zeaxanthin were found in untreated leaves which had the lowest heat tolerance. Interestingly, the amount of total xanthophyll cycle pigments decreased in PPFD60% and PPFD30%, but was almost at the same level in untreated leaves and leaves treated by PPFD12% for which heat tolerance deviated by 10.6 K. However, PPFD12% leaves had significantly higher violaxanthin levels, in agreement with the elevated pigment levels, which may lead to a higher requirement for more efficient NPQ mechanisms to be in place under heat stress. In addition, PPFD12% leaves also contained higher levels of total ascorbate, which may support xanthophyll cycle activity (see Müller‐Moulé et al. 2002).

## Conclusions

Evidence is increasing that light plays a pivotal role in the heat hardening of plants during short‐term exposure to heat, with several studies corroborating the idea that light seems to play a protective role in survival of heat stress. However, the influence of light on heat hardening of plants under long‐term heat stress is still poorly understood. Our results suggest that the responses of ROS scavenging mechanisms and pigment composition to heat hardening upon long‐term exposure are not the same as under short‐term heat exposure; and during long‐term heat exposure under low light conditions changes in pigment composition with downstream effects on thylakoid membrane stability may play a crucial role in heat hardening. We also show that different light intensities affect the hardening process in different ways, not only during long‐term heat exposure, but also during short‐term heat exposure. In untreated leaves temperatures reached 46.4°C (HHM) and heat damage due to natural overheating was observed on several individuals leaves (Appendix S5), supporting earlier assumptions that (sub)alpine plants are at high risk of experiencing critically high temperatures (Ladinig et al. 2015). This does not necessarily mean that *V. gaultherioides* will become extinct as alpine habitats are characterized by dense thermal microhabitat mosaics where plants are able to ‘escape’ (described by Scherrer and Körner 2011). However, the predicted temperature increase due to global warming in conjunction with more frequently occurring short‐term heat shocks and long‐term heat waves (IPCC 2013) will increase the pressure on *V. gaultherioides* in subalpine regions. *V. gaultherioides* may be restricted to certain ecological niches, or eventually be replaced by more competitive species.

## Author contributions

O.B. and G.N. initiated the project. O.B. built the heat‐hardening and heat tolerance testing equipment, designed and supervised the field experiments. M.K. performed the field experiments and prepared specimens for the TEM‐study. M.K. measured photosynthetic pigments, and ROS scavenging capacity. A.H. supervised electron microscopic examinations. Data were analyzed by all the authors. I.K., A.H. and G.N. advised on experimental design, and provided instruments, lab space and consumables. All authors wrote and proofread the manuscript.

## Supporting information


**Appendix S1.** Short‐term heat spell treatments during summer 2012 on Mt. Patscherkofel (1950 m a.s.l., Innsbruck, Austria) as applied in situ to Vaccinium gaultherioides plants (photographs and timing scheme).

**Appendix S2.** Temperature and solar irradiation levels during and after short‐term heat spell treatment (diagram).

**Appendix S3.** Spectrophotometric analysis of the garden fleece which was used for shading the heat‐hardening chambers (diagram).

**Appendix S4.** Long‐term heat wave treatments during summer 2012 on Mt. Patscherkofel (1950 m a.s.l., Innsbruck, Austria) as applied in situ to Vaccinium gaultherioides plants (drawing and timing scheme).

**Appendix S5.** Naturally occurring leaf damage on Vaccinium gaultherioides linked to overheating during summer 2012 on Mt. Patscherkofel (1950 m a.s.l., Innsbruck, Austria) (photographs).
