# Supplementary material for: Solar irradiation levels during simulated long‐ and short‐term heat waves significantly influence heat survival, pigment and ascorbate composition, and free radical scavenging activity in alpine Vaccinium gaultherioides
Source: Physiol Plant. 2018 Mar 13;163(2):211–30. doi: 10.1111/ppl.12686 (PMC6033156; doi:10.1111/ppl.12686)

Appendix S1. Short-term heat spell treatments during summer 2012 on Mt. Patscherkofel (1950 m a.s.l., Innsbruck, Austria) as applied in situ to *Vaccinium gaultherioides* plants.

The heat treatments were conducted by means of the Heat Tolerance Testing System (HTTS) which consists of a mobile supply unit and eight exposure chambers. The system is described in detail in: Buchner O., Karadar M., Bauer I., Neuner G. (2013) A novel system for *in situ* determination of heat tolerance of plants: first results on alpine dwarf shrubs. *Plant Methods* 2013, 9:7; doi: 10.1186/1746-4811-9-7.

A. Mobile supply unit of the HTTS containing power supplies and electronic components.

B. Exposure chambers installed in the field to apply controlled heat to *Pinus cembra* seedlings (arrow). Samples can be heat treated in situ **(1)** either in darkness (dark-mode) or **(2)** under natural solar irradiation conditions (light-mode). Freely selectable temperature ramps as well as constant temperature levels can be realized. Programming happens via special software that runs on a Netbook which is connected to the mobile supply unit (A) via LAN-Cable. Leaf transpiration can be restricted by increasing the air humidity inside the exposure chambers to 100%, and steaming up of the Plexiglas cylinders is avoided by switching on the integrated window heaters.

C. Schematic illustration showing the timing of the experimental steps and conducted measurements before and during short-term heat spell treatments. **(1)** Determination of reference values of F_v/F_m of dark-adapted untreated leaves (green circles), **(2)** performance of the in situ heat treatments in darkness (black circles) and in parallel under natural solar irradiation (yellow circles): applied target temperatures were 43, 45, 47 and 49°C (further details on the heat treatments are given in Appendix S2). **(3)** Freezing in liquid nitrogen (LN_2, -196°C) of leaves from heat treated and from untreated leaves immediately after the heat treatments for determining pigments and ascorbate levels and free radical scavenging activity (FRSA), **(4)** determination of F_v/F_m 6 h after the heat treatments, **(5)** determination of F_v/F_m and visual assessment of the leaves 3 days after the heat treatments, and calculation of heat tolerance based on F_v/F_m ($LT_{50\ Fv/Fm}$) and on the visual assessment (visual estimation method VEM: $LT_{50\ visual}$).

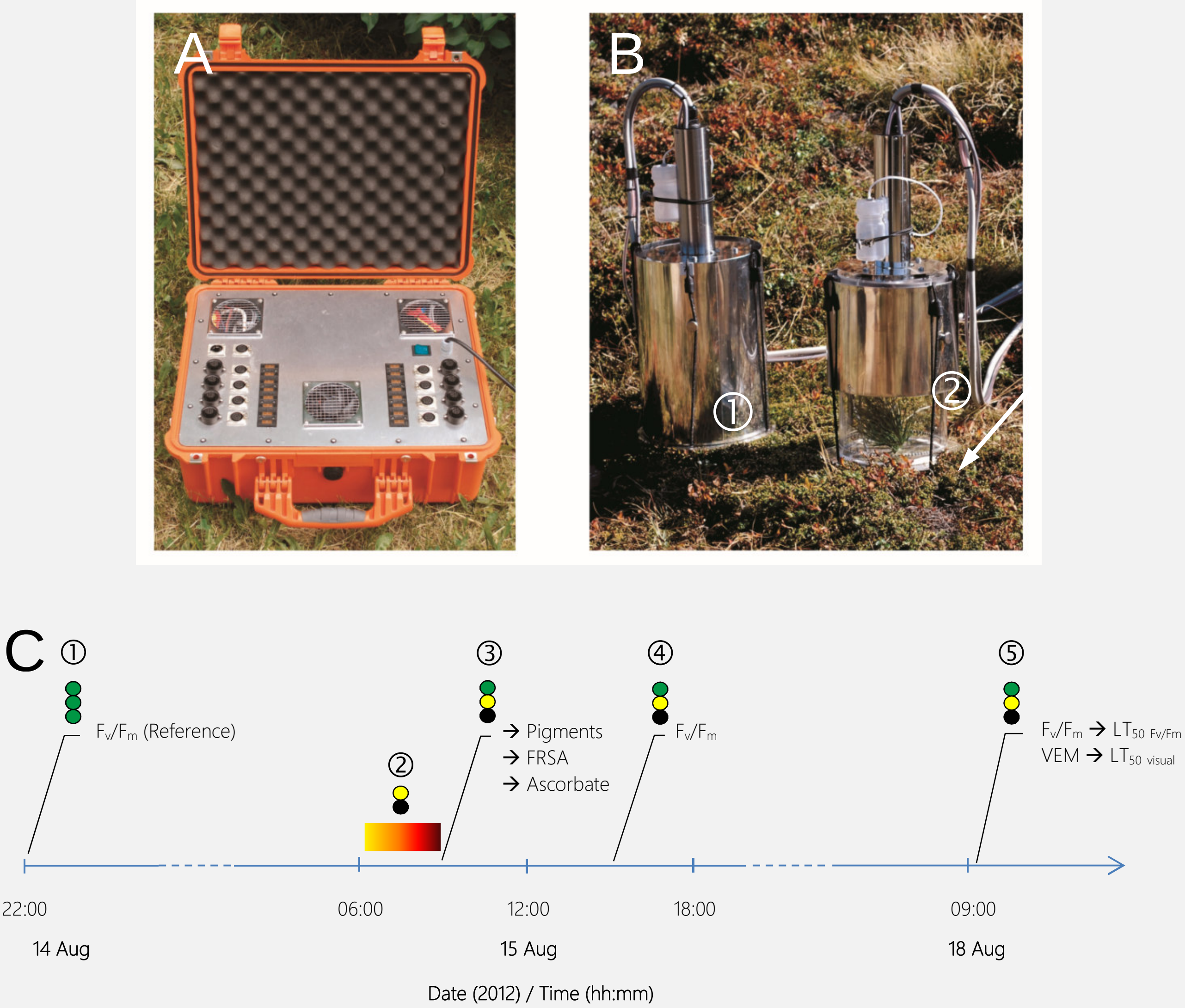

Supplement: Supplementary file 1 — Appendix S1. Short‐term heat spell treatments during summer 2012 on Mt. Patscherkofel (1950 m a.s.l., Innsbruck, Austria) as applied in situ to Vaccinium gaultherioides plants (photographs and timing scheme). [file PPL-163-211-s001.pdf]
