# Supplementary material for: Solar irradiation levels during simulated long‐ and short‐term heat waves significantly influence heat survival, pigment and ascorbate composition, and free radical scavenging activity in alpine Vaccinium gaultherioides
Source: Physiol Plant. 2018 Mar 13;163(2):211–30. doi: 10.1111/ppl.12686 (PMC6033156; doi:10.1111/ppl.12686)

Appendix S2. Temperature and solar irradiation levels during and after short-term heat spell treatment.

The experiment was conducted in situ on natural stands of *V. gaultherioides* on Mt. Patscherkofel (1950 m a.s.l.), using the Heat Tolerance Testing System (HTTS). Plants were allowed to equilibrate for 30 min at 30°C (1) and then leaf temperatures were linearly increased within 120 min from 30°C to 43, 45, 47 or 49°C (2). Thereafter these temperatures were held for 30 min (3). Treatments were either conducted under low light (maximum PPFD during (1) and (2) was 110 and 250 µmol photons m^{-2} s^{-1}, respectively) or in the darkness. Heat treatments were started before sunrise to ensure maximum physiological homogeneity of the leaves. Temperature course of leaf temperatures (green lines), air temperature (2 m) (black line) and PPFD (red line) during and after the heat treatment is shown.

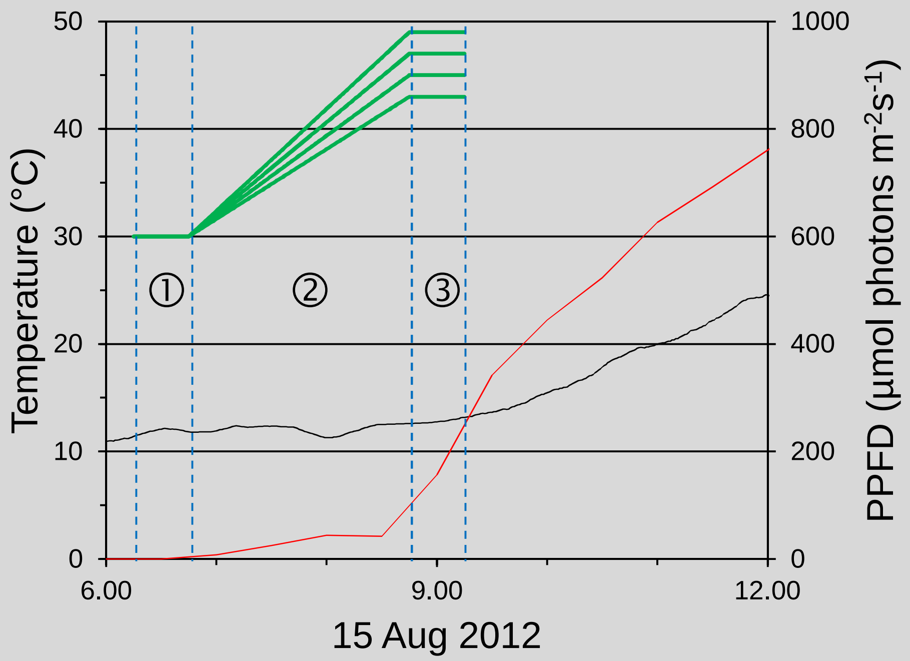

Supplement: Supplementary file 2 — Appendix S2. Temperature and solar irradiation levels during and after short‐term heat spell treatment (diagram). [file PPL-163-211-s004.pdf]
