# Supplementary material for: Solar irradiation levels during simulated long‐ and short‐term heat waves significantly influence heat survival, pigment and ascorbate composition, and free radical scavenging activity in alpine Vaccinium gaultherioides
Source: Physiol Plant. 2018 Mar 13;163(2):211–30. doi: 10.1111/ppl.12686 (PMC6033156; doi:10.1111/ppl.12686)

Appendix S3. Spectrophotometric analysis of the garden fleece which was used for shading the heat-hardening chambers.

The transmission spectrum of the garden fleece (2 layers, Windhager, Thalgau, Austria) illustrates a nearly uniform damping of the light (250 – 1000 nm) passing the fleece.

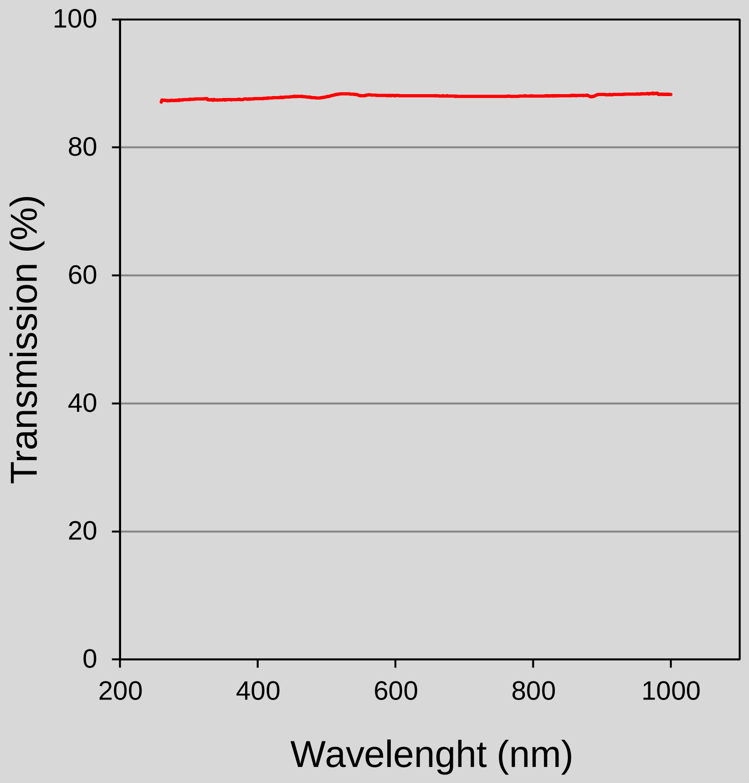

Supplement: Supplementary file 3 — Appendix S3. Spectrophotometric analysis of the garden fleece which was used for shading the heat‐hardening chambers (diagram). [file PPL-163-211-s002.pdf]
