# Supplementary material for: Solar irradiation levels during simulated long‐ and short‐term heat waves significantly influence heat survival, pigment and ascorbate composition, and free radical scavenging activity in alpine Vaccinium gaultherioides
Source: Physiol Plant. 2018 Mar 13;163(2):211–30. doi: 10.1111/ppl.12686 (PMC6033156; doi:10.1111/ppl.12686)

Appendix S4. Long-term heat wave treatments during summer 2012 on Mt. Patscherkofel (1950 m a.s.l., Innsbruck, Austria) as applied in situ to *Vaccinium gaultherioides* plants.

The heat treatments were conducted by means of 3 Heat Hardening Chambers (HHCs). Schematic illustration of a Heat Hardening Chamber (HHC) in **(A)** Front view and **(B)** Top-down view. The HHCs are made of double-walled (8 mm) Plexiglas plates. Ceramic heaters (1) which are mounted to the roof and fans (2) which are mounted through the side walls, allow for controlling the temperature inside the HHC by automatically switching on / off the heaters and fans. The control unit (not shown) to which up to 16 HHCs can be connected, is operated by special software. Inside each HHC photosynthetically active photon flux density (PPFD), air temperature and a couple of randomly selected leaf temperatures were measured in 1 s intervals. Mean leaf temperature was set to be 30°C. By shielding the HHCs with garden fleece and linen (3), which acted as a neutral filter, PPFD inside the HHCs was reduced to 60% of the solar radiation outside (PPFD60%), to 30% (PPFD30%) and to 12% (PPFD12%).

C. Timing of the experimental steps and conducted measurements. (1) Determination of reference values of F_v/F_m on leaves of untreated plants (green circles), (2) – (6) performance of the in situ heat treatments at mean leaf temperatures 30°C / 15°C (day / night) and at different mean solar irradiation levels: 60% (white circles), 30% (light grey circles) and 12% (dark grey circles) of the naturally occurring mean photosynthetically active photon flux density (PPFD). Untreated plants (100% PPFD, no temperature treatment) are indicated by yellow circles. F_v/F_m measurements and visual viability assessments on the leaves were conducted daily in the afternoon (except 4 July), and heat tolerance based on F_v/F_m ($LT_{50\ Fv/Fm}$) and on the visual assessment method (VAM: $LT_{50\ visual}$) was calculated. At the end of the heat wave treatment (6 July) leaves from heat treated and from untreated plants were immediately frozen in liquid nitrogen (LN_2, -196°C) for determination of pigment and ascorbate levels and free radical scavenging activity (FRSA).

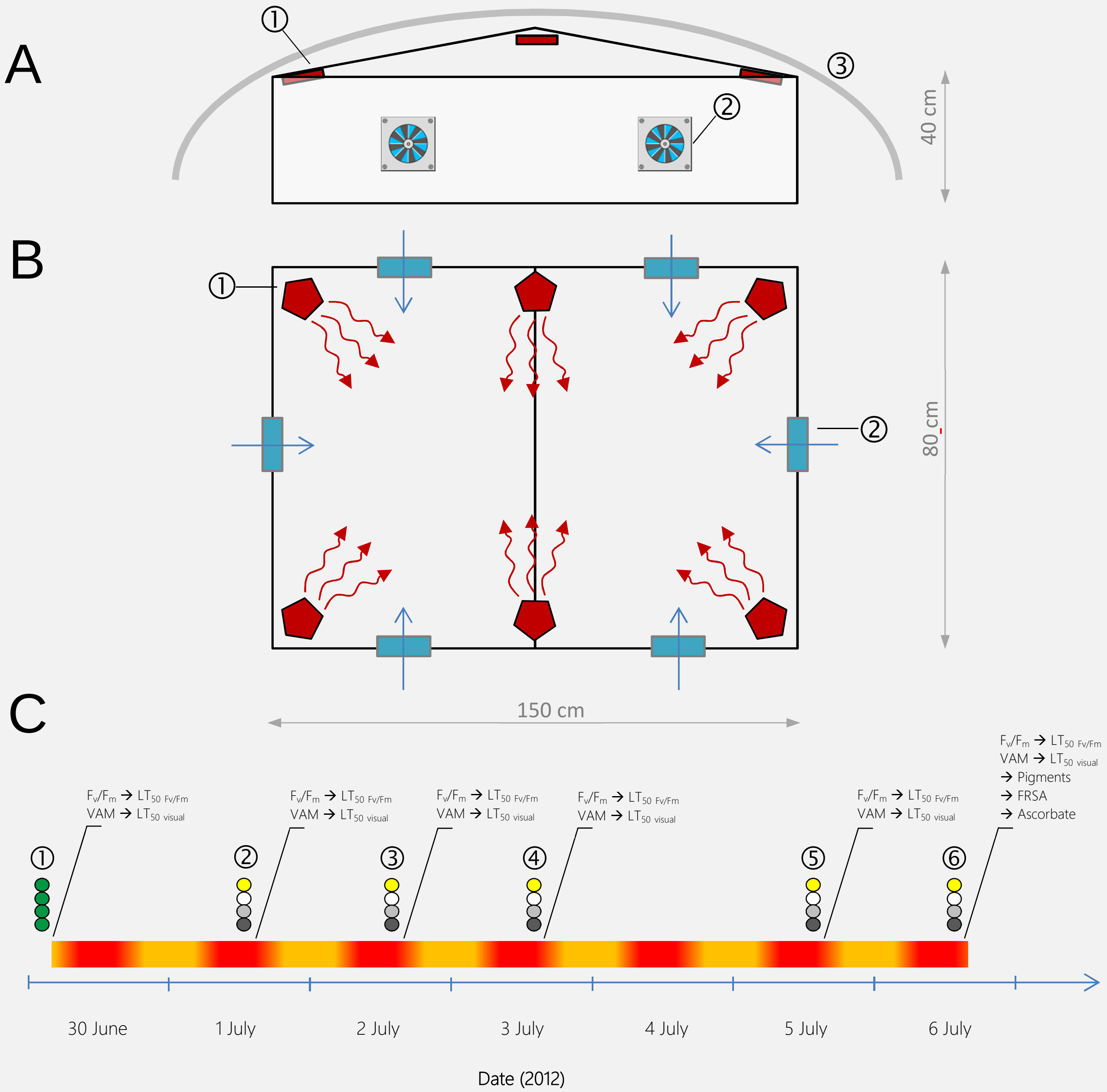

Supplement: Supplementary file 4 — Appendix S4. Long‐term heat wave treatments during summer 2012 on Mt. Patscherkofel (1950 m a.s.l., Innsbruck, Austria) as applied in situ to Vaccinium gaultherioides plants (drawing and timing scheme). [file PPL-163-211-s003.pdf]
