# Supplementary material for: Solar irradiation levels during simulated long‐ and short‐term heat waves significantly influence heat survival, pigment and ascorbate composition, and free radical scavenging activity in alpine Vaccinium gaultherioides
Source: Physiol Plant. 2018 Mar 13;163(2):211–30. doi: 10.1111/ppl.12686 (PMC6033156; doi:10.1111/ppl.12686)

Appendix S5. Naturally occurring leaf damage on *V. gaultherioides* linked to overheating during summer 2012. Necrotic leaf areas (white arrows), visible as brown discoloration, on various growing sites on Mt. Patscherkofel (1950 m.a.s.l.).

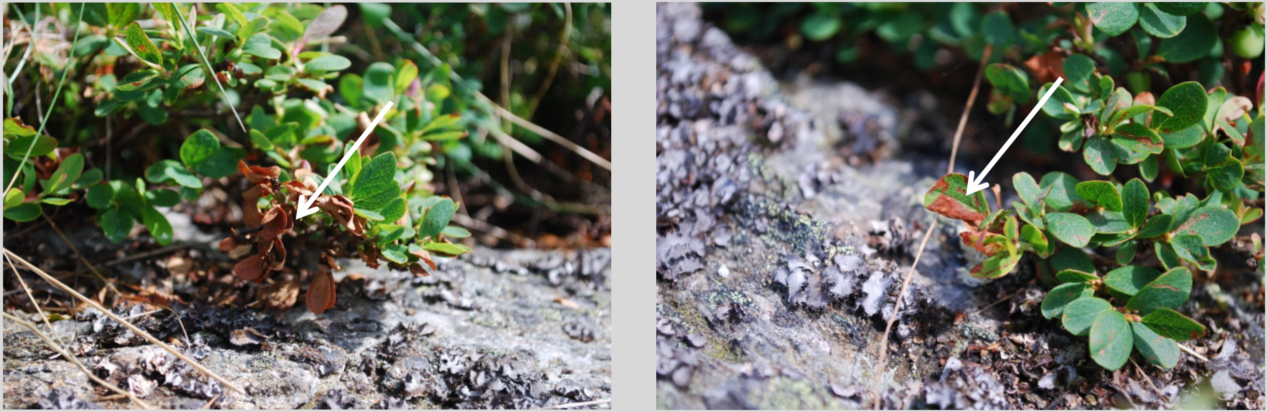

Supplement: Supplementary file 5 — Appendix S5. Naturally occurring leaf damage on Vaccinium gaultherioides linked to overheating during summer 2012 on Mt. Patscherkofel (1950 m a.s.l., Innsbruck, Austria) (photographs). [file PPL-163-211-s005.pdf]
